# Knowledge and attitude of deaf women in relation to contraceptive methods: A systematic review

**DOI:** 10.1016/j.clinsp.2024.100558

**Published:** 2025-01-27

**Authors:** Gabriela Fuster Barbosa, Edson Santos Ferreira-Filho, Lais Abdo Quintão, Laura Fernandes Berto, Patrícia Gonçalves de Almeida, Edmund Chada Baracat, Luis Bahamondes, José Maria Soares-Junior, Isabel Cristina Esposito Sorpreso

**Affiliations:** a Department of Obstetrics and Gynecology, Faculdade de Medicina, Universidade de São Paulo (FMUSP), São Paulo, SP, Brazil; b Department of Obstetrics and Gynecology, Hospital das Clínicas, Faculdade de Medicina, Universidade de São Paulo (HCFMUSP), São Paulo, SP, Brazil; c Department of Obstetrics and Gynecology, Faculdade de Ciências Médicas da Universidade Estadual de Campinas (FCM UNICAMP), Campinas, SP, Brazil

**Keywords:** Contraception, Deafness, Knowledge, Systematic Review, Woman

## Abstract

**Background:**

Communication barriers make it difficult for deaf women to access information about contraceptive methods, making them vulnerable to unplanned pregnancies.

**Objective:**

To identify knowledge and attitudes of deaf women in relation to contraceptive methods.

**Method:**

The authors conducted a systematic review of the literature, PROSPERO registry (CRD42021277635), conducted from August 2021 to April 2024. The included studies focused on knowledge and attitudes about contraception among women with deafness.

**Results:**

Twelve articles were included, with a total of 2,641 participants and those reported low knowledge about contraceptive methods. Nine studies showed withdrawal, condoms, and oral contraceptives as the best methods that deaf women know. Five studies showed low knowledge about intrauterine devices and subdermal implants. The main reasons given for abandoning the method were reproductive desire and fear of side effects. The use of hormonal contraceptives was higher among adult women and, two studies showed that among teenagers the practice of withdrawal was higher than the use of condoms. The barriers most mentioned in the eight articles for the use of contraceptives were those of communication.

**Conclusion:**

Knowledge of contraceptives among deaf women was low; however, the attitude of acceptance of the use of contraceptive methods was favorable, despite the little access to more effective methods, especially among adolescents. The factor associated with low knowledge evidenced in the systematic review was communication barriers.

## Highlights

• Knowledge was low mainly about contraceptive methods of high effectiveness.
• The acceptance of the chosen contraceptive method by deaf women was satisfactory.
• There are no intervention studies to improve contraceptive use among deaf women.

## Introduction

In health, the term “deaf” is attributed to the clinical condition of reduced sound perception classified as mild, moderate, severe, and profound.^1^^,^^2^ When hearing loss occurs before the acquisition of orality, there is difficulty in oral language literacy, and the adoption of sign language is fundamental in communication and social inclusion.^1^^,^^2^ Thus, the term “deaf” has a different conceptual perspective for people who communicate through sign language, which includes the social phenomena of the construction of the cultural identity of the deaf (deaf culture), moving away from the pathological view.^1^, ^2^, ^3^

It is estimated that about 1.5 billion people worldwide have hearing loss, and in Brazil, it is estimated almost 10 million, of which 1.8 million are women of reproductive age.^2^^,^^3^ The number of Unplanned Pregnancies (UP) among women with disabilities is high, above 60 %, when compared to the global rate which stands at 44 %, and nationally, 55 %.^4^^,^^5^ The consequences of UP among deaf women are underreported as they constitute a group with scarce attention or investment in public policies for access to contraceptive methods.^3^^,^^5^, ^6^, ^7^, ^8^, ^9^

The communication barriers, the lack of access of deaf people to preventive information, the idea that deaf people are sexually inactive, and the lack of preparation of health institutions and teams in the care of people with deafness have been reported as difficulties that the deaf population faces in accessing health. Therefore, the knowledge and attitude of deaf women towards contraception should be analyzed.^3^^,^^5^, ^6^, ^7^, ^8^, ^9^ Consequently, the objective of this review was to identify the knowledge and attitude of deaf women in relation to contraceptive methods, to elucidate the level of knowledge and attitudes toward contraception in this specific and vulnerable population, aiming for strategies to improve Sexual and Reproductive Health (SRH) for deaf women.

## Method

The present systematic review was conducted according to the standard Preferred Reporting Items for the Systematic Reviews and Meta-Analyses (PRISMA)^10^ and registered in an international registry of systematic reviews PROSPERO, CRD42021277635. To identify the knowledge and attitudes related to contraceptives among deaf women, the following keywords were used through the PICO strategy: "deaf" OR "deaf woman" OR "deaf women" OR "hearing impairment" OR "hearing loss" AND “contraception” OR “contracept*” OR “Birth Control” AND "Knowledge" OR "attitude" OR "awareness". The search was performed systematically in seven databases: PubMed, Embase, Web of Science, Scopus, Psycinfo, CINAHL and DART-E, between 23 August 2021 and 18 April 2024.

The authors used as inclusion criteria: talk about deafness focusing on knowledge and attitudes related to contraception. To contain deaf women in the sample. The authors excluded articles not published in English, Portuguese or Spanish; articles beyond the scope of this study; opinion articles, case report, narrative reviews, systematic reviews, guidelines, book or editorial type, which are based on other articles, posters and abstracts; two or more associated disabilities in a single individual, for example deaf blindness and deafness associated with mental disability, in the sample. There were no restrictions on sample size and time.

### Data selection and extraction

**Fig. 1:**
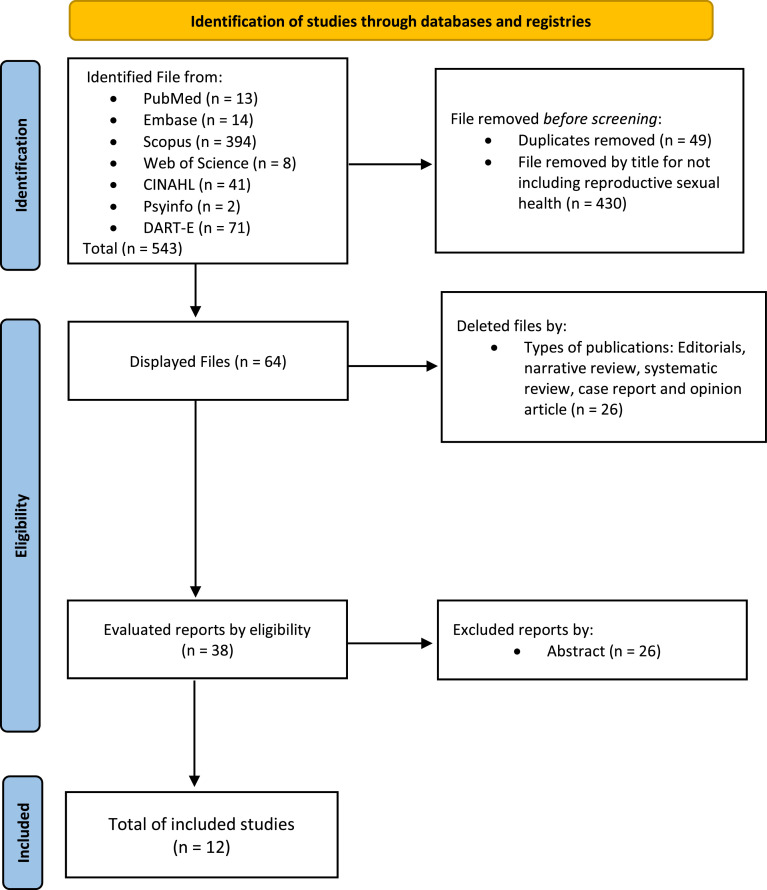
PRISMA 2020 flowchart for new systematic reviews.^16^

In the search procedure, there were three stages for screening: (1) Search for duplicate articles and removal of duplicates; (2) Reading the title, keywords and abstract of each article with subsequent exclusion according to the exclusion criteria; and (3) Detailed reading of the full texts. For the procedures of search, selection and analysis, at least two independent reviewers were assigned. In case of disagreement regarding its inclusion, the reviewers discussed the article until they obtained a final opinion. All reviewers checked the study characteristics, subject´s information and results, identifying the limitations of each study. The evaluation of the articles was carried out independently and blindly by G.F.B., L.F.B. and L.A.Q. or E.S.F.F. and supervision of searches by I.C.E.S. Fig. 1 represents the PRISMA^10^ flowchart for new systematic reviews (identification, eligibility and inclusion) (Fig. 1).

The data extracted included the study methodology, year and country of origin, number of participants, age group, sociodemographic data, knowledge about contraceptive methods, associated factors and/or attitudes in relation to contraception, the rate of contraceptive use, barriers, and facilitators for informed contraceptive use and whether there have been interventions to improve decision-making in informed contraceptive use.

The Grading of Recommendations, Assessment, Development and Evaluations (GRADE)^11^ was chosen to evaluate the evidence quality of each study, considering the limitations and quality of the study (method and execution) in an analysis of the strength of evidence. For each outcome, the evidence was classified as high, moderate, low, or very low quality. For the evaluation of the quality of qualitative studies, the COnsolidated criteria for REporting Qualitative research (CORE-Q)^12^ were chosen, which consists of a checklist of 32 items for interviews and focal groups by three domains, namely: “research team and reflexivity” (8 items), “of the study” (15 items) and “analysis and findings” (9 items).

## Results

### Search performance

The search in the consulted databases resulted in 543 studies, all accessible, with 49 duplicate articles excluded. Additionally, 430 titles were excluded because they did not address SRH, and 26 were excluded due to the type of publication. After a detailed reading of the full texts, 26 articles that did not meet the inclusion criteria were excluded. Thus, 12 articles were selected, fully read and composed the result.

### Overview

The 12 eligible articles composed a total of 2641 subjects and among those no interventional study was found, most of them performed qualitative or mixed approach (quali-quantitative). It was not possible to perform a meta-analysis due to the heterogeneity of the studies. Table 1, Table 2 present the 12 eligible articles and their classification according to the score obtained by GRADE^11^ and CORE-Q.^12^ Only two articles performed a qualitative analysis of the data,^7^^,^^9^ being classified as moderate^7^^,^^9^ according to the CORE-Q[12] evaluation. There were 10 cross-sectional quantitative articles.^6^^,^^8^^,^^13^, ^14^, ^15^, ^16^, ^17^, ^18^, ^19^, ^20^ All articles were classified, according to the score obtained by GRADE^11^ as very low quality^6^^,^^8^^,^^13^, ^14^, ^15^, ^16^, ^17^, ^18^, ^19^, ^20^ (Table 1, Table 2).

**Table 1:** Eligible articles and evaluation of quality of evidence, according to GRADE in the systematic review ‒ 2024.

Author, Year, Country	Type of study	Goal	Population (N)	Results	GRADE
			Age group (average)		
			Features sociodemographic		
Mprah, WK; Anafi, P; Yeaboah PYA, 2017, Ghana	Cross-sectional study	To evaluate the level of knowledge (deaf people) about methods of pregnancy prevention.	178 Deaf people 74/178 Deaf women 104/178 Deaf men 44 % adolescent's female and 30 % adults women 18‒50 years	35.4 % Coitus interruptus can prevent pregnancy 33.8 % A woman using oral contraceptives did not get pregnant 29.2 % Use modern contraceptive methods are protective against pregnancy 46.2 % Avoiding sex prevents pregnancy	⨁◯◯◯ Very low
Yimer, AS; Modiba, LM, 2019, Ethiopia	Cross-sectional descriptive study qualitative and quantitative	To evaluate the knowledge and practice of family planning among women with sensory impairment (deaf and blind women)	328 165/328 Deaf women 163/328 Blind women 28.57 years 59.7 % single women 7.9 % illiterate 53 % Orthodox women 3 1.7 % small business ownership 25 % unemployed	97.2 % of the interviewers have knowledge regarding Family Planning methods. The level of knowledge about modern contraceptive methods was 32.5 % The prevalence of unplanned pregnancy was 61.3 % The prevalence of contraceptive use at the time of the study was 31.1 %	⨁◯◯◯ Very low
Olajide, FO; Omisore, AG; Arije, OO; Afolabi, OT; Olajide, AO, 2014, Nigeria	Cross-sectional study	To evaluate the awareness and use of modern contraceptives among adolescents with physical disabilities at school	215 Adolescents 184/215 Hearing loss 22/215 Physical disability 9/215 Visually impaired 94/215 Women 15.5 years 65.6 % Christians and 34 %, Muslims 53.5 % primary education	33.7 % Knowledge regarding modern contraceptive method	⨁◯◯◯ Very low
Joseph, J.M.; Sawyer, R; Desmond S, 1995, USA	Cross-sectional study	To measure the sexual health knowledge and behavior of deaf students and with hearing disability at an arts university for deaf students and with hearing disability	134 University deaf Students and with hearing disability 72 % Women 28 % Men 23 years Caucasians (73 %)	The students in this sample were not particularly well-informed about sexual health and sexuality issues, were involved in high-risk sexual behaviors and were dependent primarily from colleagues for sexual health information Most students were sexually active (81 %)	⨁◯◯◯ Very low
Gurel, R; Yilmaz, DV, 2018, Turkey	Cross-sectional study	To examine the attitude of women with disabilities aged 18 to 49, to explore their attitudes towards family planning, and observe the factors that affect their attitudes	108 Women with disabilities 33/108 Hearing disabilities 53/108 Physical disabilities 14/108 Visually impaired	Women with deafness had an FPAS score of 115.61 (19.43 SD) with p = 0.888 when compared to other disabilities Attitudes toward family planning are similar across all disabilities. The sociodemographic factors analyzed did not interfere in the attitude and knowledge about reproductive planning	⨁◯◯◯ Very low
Olaleye, AO; Anoemuah, AO; Ladipo, AO; Delano, GE; Idowu, GF, 2006, Nigeria	Cross-sectional study	To explore sexual behaviors and reproductive health knowledge among youth with disabilities in a school located in Ibadan, Nigeria	103 55/103 Hearing disabilities 12/103 Speech impairment 19/103 Physical disability 10/103 Intellectual disability 7/103 Sight impairment 34/55 Deaf women 10‒25 years 70 % Christian 30 % Muslim	33 % were informed about contraceptives 64 % knew the male condom 24 % knew about the female condom	⨁◯◯◯ Very low
Mekonnen, AG; Bayleyegn, AD; Aynalem, YA; Adane, TD; Muluneh, MA; Asefa, M, 2020, Ethiopia	Cross-sectional study	To assess the level of knowledge, attitude and practice of family planning and associated factors among persons with disabilities in the northern zone of Shewa, Amhara regional state, Ethiopia	397 57/397 Hearing impaired 13/397 Partial mental impairment 94/397 Visual impairment 197/397 Impaired mobility 53/397 Multiple impairments 173/397 women 27.7 years 48.1 % single 48 % Completed the elementary school 41.1 % unemployed	46 % knew family planning methods Best known method: injectable (74.8 %) 24.5 % used some family planning method Subjects who completed university education were 7 times more likely to have a good knowledge of family planning methods than uneducated subjects (AOR = 7.23; 95 % CI 2.28, 22.06)	⨁◯◯◯ Very low
Sawyer, RG; Desmond, SM; Joseph, JM, 2013, USA	Cross-sectional study	To compare two populations of university students, listeners and audit disabilities or deaf, relating to sexual knowledge, behavior and sources of information	305 181/305 Hearing 124/305 Hearing impaired 89/305 Hearing impaired women College students 18 years of age and older 232 Caucasians	Listening students had more knowledge about SSR than deaf/ hearing deficient students. Deaf/ hearing deficient had greater use of coitus interruptus and condoms as a contraceptive method	⨁◯◯◯ Very low
Qi W, Li H, Lian Q, Zuo X, Yu C, Lou C, Tu X, 2023, China	Cross-sectional study	To understand the SRH knowledge and its associated factors, as well as barriers and preferences in accessing sexuality-related information among unmarried youth with different types of disabilities in both urban and rural areas in China	473 207/473 Hearing disability 158/473 Visual disability 108/473 Physical disability 106/207 deaf women 15‒24 years	To assess knowledge, three variables were analyzed: 1. contraception 2. physiology 3. sexuality and STIs. Regarding knowledge of contraception: deaf 35.7 vs. visually impaired 42.9 vs. physically impaired 28.6 p < 0.001. Total knowledge of the three proposed variables: deaf 36.4 vs. visually impaired 48.5 vs. physically impaired 36.4 p < 0.001.	⨁◯◯◯ Very low
Beyene GA, Munea AM, Fekadu GA, 2019, Ethiopia	Cross-sectional study	To assess modern contraceptive use and associated factors among women with disabilities in Gondar city, Ethiopia	267 Women disabilities 32/267 Hearing impairment 101/267 Visual impairment 133/267 Limb defects 33.37 years	21.7 % had ever used modern contraceptives 15.8 % (*n*=35) were current users of modern contraceptives Among them, the dominant modern contraceptive method was the injectable method (65.70 %), followed by COC + condoms (8.60 %)	⨁◯◯◯ Very low

**Table 2:** Eligible articles according to the systematic review. Evaluation of quality of evidence, according to CORE-Q, 2024.

Author, Year, Country	Type of study	Goal	Population (N)	Results	CORE-Q
			Age group (average)		
			Sociodemographic Characteristics		
Mprah, WK, 2013, Ghana	Qualitative and quantitative with Focus groups	To investigate the level of knowledge and use of contraceptive methods among deaf people in Ghana to aim understanding their contraceptive behavior and to improve the access	179 Focus group: 10 male deaf adults; 9 women deaf adults; 7 Hearing 152/179 deaf people students, men and women 18‒60 years	From 13 listed methods presented, only three were known to 70 % of adults and 60 % of students. The level of knowledge of the remaining nine methods was low The most well-known methods among deaf people were the traditional methods and among the modern methods only oral contraceptives, injectables and condoms	17/32 Moderate
Burke, E.; Kebe, F; Flink, I; Reeuwijk, M; May, A, 2017, Senegal	Qualitative “peer-to-peer”	Understand the barriers and facilitators the youngest with disabilities face when accessing SSR services. Explore (1) the expressed needs and SSR vulnerabilities of youngest with disabilities; and (2) their experiences of accessing SRH services, including the challenges facing to access these services	144 Young people with disabilities 50 interviewees: 14 Hearing disability (6 Deaf women); 21 Visual impaired; 15 Physical disability Description of the population addressed in the focus groups was not detailed 18‒24 years	Better knowledge of contraceptive methods in the groups with hearing disability – the condom and the oral contraceptives were the most cited. Young women have demonstrated conservative and critical attitudes toward sex and contraceptive use outside of marriage	16/32 Moderate

There were eight publications from North African countries,^6^, ^7^, ^8^, ^9^^,^^14^^,^^15^^,^^17^^,^^20^ followed by two in the United States (USA),^16^^,^^18^ Turkey^13^ and China.^19^ Regarding the year of publication, it ranged between 1995 and 2021, with a higher number of publications after 2010 (seven publications). The most frequent publications found in the searches were performed in countries located in the north of the African continent.^6^, ^7^, ^8^, ^9^^,^^14^^,^^15^^,^^17^^,^^20^

Of the 12 eligible articles, none addressed this theme including only deaf women in the sample. Ten articles presented in the discussion a comparative knowledge between deaf and hearing people or between the different existing disabilities (motor, visual and intellectual).^6^^,^^9^^,^^13^^,^^14^^,^^15^^,^^17^^,^^19^^,^^20^ There were three studies that addressed the identification of knowledge about SRH.^7^^,^^15^^,^^18^

Approaches related to barriers to access, sexual behavior and knowledge about prevention of sexually transmitted infections were identified.^8^^,^^9^^,^^16^^,^^18^ Studies have shown low knowledge about contraceptive methods among deaf women, especially about more effective contraceptives, and one article showed a high risk for UP among deaf women.^6^ In three studies, it was shown that among deaf women the most well-known methods were barriers and short-term methods.^7^^,^^15^^,^^17^ The rate of use of contraceptive methods was higher among barrier and short-term methods.^6^^,^^13^^,^^15^^,^^16^^,^^20^ In Table 3 the authors presented the relationship of the main findings of the categories sought in the searches, knowledge about contraceptive methods, associated factors and/or attitudes towards contraception, rate of contraceptive use, and barriers and facilitators for use of contraceptives. Also, whether there were interventions to improve decision-making in the use of contraceptives (Table 3).

**Table 3:** Knowledge associated factors/attitudes and rate of contraceptive use among deaf women in eligible studies, 2024 review.

Author(s) of the study, year	Knowledge regarding contraceptive methods	Associated factors/attitudes toward contraception	Rate of contraceptive use	Barriers and facilitators for the informed use of contraceptives	Interventions to improve decision-making in the informed use of contraceptives
Mprah, WK, 2013	**TRADITIONAL METHODS** ADULT WOMEN: Coitus interruptus 52.4 % (*n*=11) rhythm method/ periodic abstinence: 76.1 % (*n*=16); ADOLESCENTS: Coitus interruptus 43.2 % (*n*=19) rhythm method/ periodic abstinence: 52.3 % (*n*=23); **MODERN METHODS** ADULT WOMEN: Oral contraceptives 47.6 % (*n*=10) Injectables 71.4 % (*n*=15) Condoms 61.9 % (*n*=13) Emergency contraceptive 47.6 % (*n*=10) Intrauterine device (IUDs) 52.3 % (*n*=11) Implants 23.8 % (*n*=5) Spermicides 28.6 % (*n*=6) Female sterilization 23.8 % (*n*=5) Male sterilization 28.6 % (*n*=6) ADOLESCENTS: Oral contraceptive 54.5 % (*n*=24) Injectables 54.5 % (*n*=24) Condoms 52.3 % (*n*=23) Emergency contraceptive 56.8 % (*n*=25) Intrauterine device (IUDs) 22.7 % (*n*=10) Implants 38.6 % (*n*=17) Spermicides 22.7 % (*n*=10) Female sterilization 22.7 % (*n*=10) Male sterilization 15.9 % (*n*=7)	Reasons for contraceptive use: Fear of AIDS or HIV: Adult women 47.1 %; Students 62.9 % Fear of pregnancy: Adult women 62.5 %; Students 21.4 % Fear of Other Sexually Transmitted Infections: Adult women 16.7 % Students 28.6 %	Using any contraceptive: Women 32.3 %; Students 35.9 %; Adults 46.7 %	Communication barriers	Not reported
Burke, E; Kébé, F; Flink, I; Reeuwijk, M; May, A, 2017	Poor knowledge of contraceptives was identified among young people with hearing deficiency – condoms and pills were most often cited	Young women presented conservative and critical attitudes about sex and contraceptive use outside of marriage	35 % (8/23) reported never use of contraception	Myths regarding sexuality Religion/Faith as a barrier Sources of advice: friends were the main confidants Main barriers to access to SSR services: lack of confidentiality, anonymity and distance Other barriers: parental attitude and communication barriers Dependence of family members to follow in the medical appointments and health services.	Not reported
Yimer, AS; Modiba, LM, 2019	Deaf women were inclined to demonstrate comprehensive knowledge about contraceptives when compared to blind women (p = 0.01)	Less tendency to use any modern contraceptive method: young woman (15‒24 years old), single, with little knowledge, deaf and with low self-perception Reasons raised up for contraceptive abandonment: fear of collateral effects (41.2 %), infrequent sexual intercourse (29.4 %), lack of knowledge (23.5 %) and desire to get pregnant (21.6 %) 32.4 % of family planning users used contraception to delay the first pregnancy 13 (12.7 %) 1/4 used contraceptive methods for fear of forced sexual intercourse or rape	31.1 % use any contraceptive method 44.2 % short duration methods (oral contraceptives, 11.8 %; injectables, 15.7 % and male condom 16.7 %) 51 % implants 5 % IUDs 75.6 % never used condoms	The most common source of information: friends/colleagues The family can exert a positive or negative influence in the contraceptives access Most current users (78.4 %) obtained contraceptives in public health units Deaf women are less likely to use family planning services (AOR = 6.4; 95 % CI 3.40, 12.01)	Not reported
Olajide, FO; Omisore, AG; Arije, OO; Afolabi, OT; Olajide, AO, 2014	38 % already heard about modern contraceptive methods Presents knowledge regarding contraceptive method: 29.6 % of women (p = 0.026) **Knowledge** (*n*=82): Male condom 26 %; Female condom 19.1 %; Injectable 20.5 %; COCs 15.8 %; IUDs 9.8 %; Spermicides 6.9 %	The interviewers in late adolescence phase were more aware of modern contraceptives than those in other age groups (p < 0.001) 33.7 % of hearing disability knew some modern contraceptive, compared to 59.1 % with limb disability and 77.8 % with visual disability (p = 0.003) The male condom was the most used contraceptive (28 %)	**Current use** (*n*=35): Male condom; 28.6 % (*n*=10); Female condom; 14.3 % (*n*=5); Injectable 5.7 % (*n*=2); COCs 8.6 % (*n*=3); IUDs 0 % (*n*=0); Spermicides 2.9 % (*n*=1)	The most raised up source of information was television and radio (79.2 %) and the least cited were the internet (18.3 %)	Not reported
Joseph, JM; Sawyer, R; Desmond, S, 1995	Not addressed	Reasons for not using a condom in the last sexual intercourse: Having a steady partner (51 %) and not like the method (25 %) Students who reported a steady partnership were the least likely to have used a condom in their last sexual intercourse (24 %)	34 % reported having used a condom in their last sexual intercourse The most used contraceptives were coitus interruptus (45 %), condoms (34 %) and oral contraceptives (17 %)	The most raised up source of information was friends (81 %), magazines (71 %), television (59 %) and doctors (59 %) Female students were more likely to seek television 64 % and nurses 86 % as sources of health information	Not reported
Gurel, R; Yilmaz, DV, 2018	Not addressed	50 % of women who use family planning methods use one method for 13 months or more and 57.7 % have stopped using the methods for any reason (for example, the desire of get pregnant) Women who married at age 26 or older have a more positive attitude toward family planning when compared to women who marry between the ages of 19 and 25	36.3 % of them use condoms, 33.3 % use IUDs, 21.2 % use oral contraceptives	16.7 % of women with disabilities receive information about family planning methods, 72.2 % of women with disabilities who receive information were informed by an obstetric nurse and 11.1 % of them were informed by a doctor	Not reported
Olaleye, AO; Anoemuah, AO; Ladipo, AO; Delano, GE; Idowu, GF, 2007	41 % of the interviewers were aware of at least one contraceptive (other than a condom) 33 % of the hearing impaired were aware of contraception methods Women presented less knowledge regarding contraceptive methods (14 %) than men (27 %) 64/103 knew the male condom 24/103 knew the female condom	From 36 sexually active, 17 reported they began sexual activities by themselves (with no defined reason), 9 were influenced by colleagues, 5 reported being “experimenting” sex, 4 were raped, while 1 reported had the first sexual activity for monetary gain five and four interviewees stated that they practiced masturbation to avoid unplanned pregnancy and STI/HIV, respectively	From 23 interviewees who had used some contraceptive method (including condoms), 19 reported have used contraceptive method in the last sexual intercourse Six interviews were consistent condom users (using condoms in all sexual intercourse) The male condom was the most used contraceptive method	Lack of access to educational programs Lack of access to reproductive health services, such as STI treatments and services related to pregnancy and/or contraception health services	Not reported
Mekonnen, AG; Bayleyegn, AD; Aynalem, NOW; Adane, TD; Muluneh, MA; Asefa, M, 2020	46 % of the subjects presented knowledge regarding contraceptive methods COCs: 62.8 % Condoms: 54.4 % Injectable: 74.8 % Implants: 56.6 % IUDs: 38.9 % Sterilization (male and female): 27.4 % Amenorrhea lactational (LAM): 24.1 % Rhythm method 28.8 % Periodic abstinence: 39.4 % Coitus interruptus: 18.1 %	Subjects with elementary education: 3 × more chance to have great knowledge regarding contraceptive methods than participants without education (AOR = 3.31; 95 % CI 1.37, 7.59) Subjects with great knowledge of contraceptive methods: 1.6 × more likely to use any type of method than those with less knowledge (AOR = 1.61, 95 % CI 1.27, 16.24) 55 % demonstrates a positive attitude towards contraception	24.4 % reported current use of some contraceptive method, 77.6 % of these samples are female	Source of information: television/radio 69.1 %	Not reported
Beyene GA, Munea AM, Fekadu GA, 2019	Not addressed	WWDs who had limb defects/physical impairment were 6 (AOR = 5.9, 95 % CI 1.21‒28.80) times more likely to use modern contraceptives than those who had hearing impairment	Type of contraceptive currently used: 65 % Injection; 8 % Condom & COC; 6 % Condom; 6 % COC; 6 % Implant; 6 % Condom & implant; 3 % IUD	42.9 % were not satisfied with the services provided by health professionals. 48 % reported that the attitude of healthcare professionals was not good. 30 % said healthcare institutions were inconvenient. 18 % mentioned that they did not receive special care. 33 % believed that the inconvenience of healthcare institutions was the main obstacle to the use of modern contraceptives.	Not reported

### Knowledge of contraceptive methods among deaf women

Ten studies addressed specific issues regarding behavioral, barriers, short-acting, long-acting, and permanent contraceptive methods.^6^^,^^7^^,^^9^^,^^13^, ^14^, ^15^, ^16^, ^17^, ^18^^,^^20^ Barrier methods such as condoms were the best known in six articles compared to other reversible contraceptive methods (pills, injectables, Intrauterine Devices [IUDs]) and included comparison groups with people without disabilities or other disabilities.^9^^,^^13^, ^14^, ^15^, ^16^^,^^20^ Studies that had people without disabilities or other disabilities (visual and physical) as a comparative group showed that deaf women had less knowledge about more effective contraceptive methods.^9^^,^^13^, ^14^, ^15^^,^^20^ In two of these studies, it was shown that the male condom was better known than the female condom among deaf women.^15^^,^^18^

Fertility awareness contraceptive methods like coitus interruptus and the rhythm method were better known among deaf women in three articles when compared to other reversible contraceptive methods and to deaf men^7^^,^^16^ or people without disabilities.^18^ Among the short-acting contraceptive methods, injectables and oral contraceptives were better known among deaf women, although, the use of oral contraceptives was higher than injectables.^6^^,^^7^^,^^9^^,^^13^^,^^15^, ^16^, ^17^ There was limited knowledge of Long-Acting Reversible Contraceptive methods (LARCs), such as IUD and the subdermal implant, with few mentions by participants in focus groups. Additionally, a lower frequency of LARC use was shown among deaf women compared to other reversible contraceptive methods.^7^^,^^9^^,^^15^^,^^20^

### Associated factors and attitudes in relation to contraception

In three articles it was reported that the reasons for the use of contraceptives among adult women are associated with avoiding UP and in adolescents, the use of contraceptive methods was also associated with the requirement of avoiding Human Immunodeficiency Virus (HIV).^6^^,^^7^^,^^13^ Another reason shown for use was the increase in the inter-pregnancy interval.^6^ The most reported reason for abandoning the method was fear of side effects.^6^^,^^13^ Among deaf university students, a steady partner was reported as a reason for not using condoms, a method considered as not unnecessary by deaf women.^16^

Condoms have been shown to be the most well-known method used among sexually active deaf adolescents.^15^^,^^16^ However, a US-based study showed that the practice of coitus interruptus was higher than the use of condoms among sexually active deaf adolescents (45 % coitus interruptus vs. 34 % condom, *n*=108).^16^ Contraceptive use was lower among young women aged between 15 and 24 years, deaf, single, with less knowledge, and less self-perception when compared to women with visual impairment.^6^ Fear of sexual violence or rape was reported as a reason for using some contraceptive methods by a quarter of interviewed deaf women.^6^^,^^14^

### Barriers and facilitators for informed use of contraceptives

The most common barriers reported in eight articles for the informed use of contraceptives were communication barriers, such as difficulty reading the written language spoken in the country and the services not having sign language interpreters.^6^^,^^7^^,^^9^^,^^14^, ^15^, ^16^, ^17^^,^^19^ Studies conducted with women with different types of disabilities, including deafness, showed that the most sought-after sources of information about contraception were people outside the family, such as friends/colleagues, and media (television, internet, radio, magazines).^6^^,^^9^^,^^14^, ^15^, ^16^, ^17^^,^^19^ Other barriers reported were financial, lack of access to SRH services, myths and convictions related to sexuality, dependence of family members, and family attitudes.^6^^,^^7^^,^^9^^,^^14^, ^15^, ^16^, ^17^

## Discussion

The literature regarding this theme is scarce, predominantly composed of observational studies with low-quality of evidence and focusing on communication barriers as the reason for the lack of knowledge of issues related to health. It was found low knowledge about contraceptive methods among deaf women in the studies when compared to women without disabilities or with other types of disabilities (visual and physical). The attitude in relation to contraception was positive regarding the adherence of deaf women to contraceptive methods even in the presence of low knowledge; however, with a higher rate of use of less effective methods such as barrier methods. The main reason for the low access to contraceptive methods was the communication barrier, making it almost impossible to reach information with healthcare professionals. It was also shown that deaf women, like women with other disabilities, seek information about contraception more through alternative sources than through healthcare professionals or families.

The low cost and lower complexity in acquiring condoms is a possibility of improving knowledge and acceptability of this method.^6^^,^^9^^,^^14^^,^^21^, ^22^, ^23^, ^24^ The same attitude of adherence to condoms among deaf women was not seen in a Brazil-based study conducted with female listeners, who presented higher adherence to oral contraceptives and permanent methods.^21^ Even among females listening in a situation of vulnerability (low years of schooling, residents in rural areas and without health insurance), adherence was higher to permanent methods in comparison to condoms.^21^^,^^22^ In addition, the female condom was less known and had lower adherence among female listeners in a study conducted in Ghana, suggesting that the knowledge and attitude of acceptance to the use of female condoms were similar between deaf and female listeners considering that culture influenced regardless of the presence of disability.^14^^,^^15^^,^^22^

In the group of short-term methods, deaf women presented higher use of oral contraceptives, similar to non-disabled women.^6^^,^^7^^,^^9^^,^^21^ A study conducted on the use of LARCs in Latin America and the Caribbean showed a higher rate of short-term contraceptive methods, especially oral contraceptives, compared to LARC and permanent contraception among non-disabled women.^31^ Studies reported an idea that deaf women search for information about contraception among lay people, to avoid meeting with healthcare professionals due to communication difficulties.^6^^,^^7^^,^^9^^,^^15^^,^^16^^,^^24^, ^25^, ^26^ Furthermore, a study in Brazil showed that most women obtain contraceptives over the counter. These results were in agreement with the findings which showed that SARC is more accessible than LARC.^21^^,^^27^, ^28^, ^29^, ^30^, ^31^, ^32^, ^33^

In a study that explored the barriers to contraceptive decision-making among women with disabilities, several women reported that they could not actively participate in the choice of contraceptive, received limited information, or were charged with a contraceptive chosen by others.^9^^,^^24^ A comparative study of women with and without disabilities showed that the proportion of LARCs use was lower among women with disabilities than among women without disabilities (5.4 % vs. 9.3 %, p = 0.005), which could be a result of existing myths regarding sexuality of people with disabilities and to the difficult access that discourages the choice, like LARCs which needs clinical visits for placement and lack of healthcare providers able to provide care for those people including a lack of a sign language interpreter.^9^^,^^25^ Policies regarding the inclusion of people with disabilities were created to address disabilities in a general context, but there are still challenges, and inequities in access to the health system, especially for deaf individuals.^3^^,^^5^^,^^9^^,^^24^, ^25^, ^26^

The reasons for using contraceptives among deaf women were to avoid or spacing pregnancy together to avoid infections such as STI/HIV/AIDS. The fear of sexual violence was raised as a reason for contraceptive use among deaf women.^6^^,^^14^

One study reported that women with disabilities were surprised to experience side effects of the contraceptive and reported frustration due to a lack of anticipated information which is common among deaf women.^24^

Among women who were deaf, aged less than 24 years and older than 35 years, single, there was a lower chance of using a more effective contraceptive method, which can be attributed to the lack of dialogue about sexuality, initially inside their families.^6^^,^^9^^,^^14^ In the USA, some women with disabilities related how sex and contraception were taboo inside their families during their youth.^24^ Some deaf women reported that their family had never addressed the subject of sexuality and birth control, discovering the existence of the topic after the age of 20.^24^

Education level is another factor suggested by studies that influence the choice of contraceptive methods.^6^, ^7^, ^8^, ^9^^,^^13^, ^14^, ^15^, ^16^, ^17^, ^18^, ^19^, ^20^, ^21^^,^^23^^,^^24^ Deaf women, presented a characteristic of low years of schooling.^6^, ^7^, ^8^, ^9^^,^^13^, ^14^, ^15^, ^16^, ^17^, ^18^, ^19^, ^20^

It is important to highlight that deaf women presented as a characteristic, in this review, an unfavorable position in the labor market and low income. The rate of use of barrier and Fertility Awareness Methods (FAM) is high due to financial access to these methods, as well as they are of low complexity and do not require a medical prescription and are present in the deaf culture.^7^^,^^9^^,^^24^^,^^26^

The choice of highly effective contraceptives is influenced by myths that surround the listening population due to the evident communication barrier, which keeps the deaf woman dependent on the listener to acquire information.^6^^,^^7^^,^^9^^,^^14^, ^15^, ^16^, ^17^^,^^26^, ^27^, ^28^, ^29^, ^30^, ^31^ The deaf population depends on lay friends and relatives for information, leading sometimes to inadequate information for decision-making.^21^^,^^24^^,^^32^

The highlight of this review showed that the acceptance and attitude regarding contraceptive methods among deaf women is satisfactory considering adherence to the contraceptive method of choice, even if the method is of low efficacy. The knowledge was low mainly about contraceptive methods of moderate and high effectiveness. To date, no intervention studies to improve knowledge and informed use of contraceptive methods among this population have been published.

### Limitations

The scarcity of prospective studies with deaf women and the standardization of methods, forbid performing a meta-analysis about the theme. The difficulty found in the description of the methods and the grouping of disabilities did not allow an analysis of an exclusively deaf population.

## Conclusion

The uptake of contraceptive methods was favorable and the knowledge among deaf women was considered low in this review. Factors associated with low knowledge regarding contraceptive methods include communication barriers and lack of information/access to more effective methods, reinforcing the importance to of reducingmmunication barriers to improve access and informed decision-making as well as to expand contraceptive methods for populations with disabilities.

## Declaration of generative AI and AI-assisted technologies in the writing process

During the preparation of this work, the author(s) did not use generative AI and AI-assisted technologies.

## Declaration of competing interest

The authors declare no conflicts of interest.
